# Ultrahigh-speed point scanning two-photon microscopy using high dynamic range silicon photomultipliers

**DOI:** 10.1038/s41598-021-84522-0

**Published:** 2021-03-04

**Authors:** Vincent D. Ching-Roa, Eben M. Olson, Sherrif F. Ibrahim, Richard Torres, Michael G. Giacomelli

**Affiliations:** 1 Department of Biomedical Engineering, University of Rochester, 207 Goergen Hall, BOX 270168, Rochester, NY 14627 USA; 2 Department of Laboratory Medicine, Yale University, New Haven, CT USA; 3 Department of Dermatology, University of Rochester Medical Center, Rochester, NY USA; 4 Rochester Dermatologic Surgery, PC,, Victor, NY USA

**Keywords:** Biophotonics, Confocal microscopy, Multiphoton microscopy, Fluorescence imaging, Optical sensors

## Abstract

Conventional two-photon microscopes use photomultiplier tubes, which enable high sensitivity but can detect relatively few photons per second, forcing longer pixel integration times and limiting maximum imaging rates. We introduce novel detection electronics using silicon photomultipliers that greatly extend dynamic range, enabling more than an order of magnitude increased photon detection rate as compared to state-of-the-art photomultiplier tubes. We demonstrate that this capability can dramatically improve both imaging rates and signal-to-noise ratio (SNR) in two-photon microscopy using human surgical specimens. Finally, to enable wider use of more advanced detection technology, we have formed the OpenSiPM project, which aims to provide open source detector designs for high-speed two-photon and confocal microscopy.

## Introduction

Photomultiplier tubes (PMTs) are widely used for fluorescence microscopy due to their combination of high gain, high bandwidth, large photosensitive area, very low dark current, and single-photon sensitivity. However, vacuum tubes are a core component of PMTs, making them expensive and easily damaged, and resulting in relatively low saturation powers compared to silicon sensors. In addition, available photocathode materials have limited quantum efficiency (QE), especially around 650–850 nm wavelengths increasingly used for deep tissue imaging. For imaging at low speeds, these limitations are less apparent because photons can be accumulated over a longer interval of time per pixel, reducing the need for high dynamic range and partially mitigating the low QE. However, applications such as tissue clearing frequently require imaging large volumes of tissue, making low speed imaging impractical for all but the smallest specimens^1^. Similarly, high imaging rates are critical for clinical applications such as real-time surgical assessment where large areas of tissue must be accessed rapidly^2,3^.

At higher imaging speeds, the maximum shot noise limited SNR of PMTs is fundamentally restricted because a finite photon detection rate imposes a maximum number of photons per pixel that cannot be exceeded without pushing the detector into saturation or damaging it. For example, a PMT with a saturation power of 2 billion photons per second imaging at a pixel rate of 10 megapixels/s (MP/s) has a theoretical maximum SNR for an otherwise noiseless detector of 14.1. In reality, the actual SNR will be lower because the electron multiplication process in a PMT results in additional electronic shot noise, called excess noise, which reduces the shot-noise limited SNR below the level predicted from the photon shot noise alone^4^. Photon counting is insensitive to variations in electron multiplication and so eliminates excess noise^5^, but the need to sequentially detect photons further reduces dynamic range, resulting in even lower maximum imaging speed for a given SNR. For example, a recent manuscript demonstrated high-speed, multipoint-scanning two-photon imaging using photon counting PMTs and 16 parallel detectors to improve throughput, but had a maximum shot-noise limited SNR of 3.6 when operated at 77 MP/s^6^. As a result, extensive computational noise reduction^6^ or averaging^7^ may be required to extract usable images. As such, imaging systems using PMTs face a severe tradeoff between imaging speed and SNR.

As an alternative, light sheet microscopy has increasingly been used because of its combination of high imaging speed and high SNR^8^. In contrast to a point scanning confocal or two-photon system, light sheet microscopy uses an array of millions of parallel pixels integrated into a CMOS image sensor. Because detection happens completely in parallel (global shutter) or in parallel rows of pixels (rolling shutter), the maximum photon detection rate is the individual pixel’s full well capacity times the number of pixels times the frame rate, a product which is typically on the order of trillions of photons per second. Furthermore, as there is typically no electron multiplication process, there is no excess noise. While overcoming the limited SNR and imaging speed of point scanning systems, light sheet microscopes have increased sensitivity to light scattering, reduced axial resolution and a reduced ability to image deep into tissue^9^. Thus, current detector technologies force a tradeoff between resolution and resilience against scattering on one hand, and imaging throughput and SNR on the other.

Silicon photomultipliers (SiPMs) have emerged as a promising alternative to PMTs for single photon sensitive detection in applications such as LIDAR due to their low cost, high sensitivity and extreme durability^10^. In a SiPM, a parallel array of avalanche diodes is configured in Geiger mode, wherein each diode is biased beyond its breakdown voltage resulting in a single photon triggering an avalanche that is quickly quenched. This configuration generates a fast binary signal upon photon detection. As a parallel array of photon counters, the excess noise associated with electron multiplication is nearly eliminated, while the high parallelism effectively produces an analog output. In contrast to conventional photon counting, where one or a small number of detectors results in rapid photon pile up and correspondingly limited dynamic range, SiPMs can have tens of thousands of parallel detectors with enormous practical dynamic ranges^11^. In addition, the use of silicon enables very low cost, high QE at longer wavelengths, rapid recovery from saturation, and very high resistance to optical damage, making them attractive for biomedical imaging.

Recently, we designed a simple detector module around a current generation SiPM and evaluated it for use in laser scanning fluorescence microscopy^12^. Our results showed that compared to high-end GaAsP or GaAs PMTs typically used in two-photon microscopy, the sensitivity of the SiPM was higher due to very low excess noise, but at low imaging rates dark counts limited SNR. Conversely, at high imaging rates as used for resonant scanning, we showed theoretically and experimentally that dark counts do not make a significant contribution to total system noise. In this manuscript, we introduce improvements in SiPM electronics that greatly extend the detector dynamic range and bandwidth while reducing costs. We demonstrate that this improved design can enable dramatically faster imaging speeds without loss of SNR as compared to state-of-the-art GaAsP PMTs. To encourage further development in high throughput imaging, we are providing our designs under open source license. Finally, we consider the fundamental imaging rate limitations for conventional two-photon microscopy using point scanning.

## Methods

### Extending the dynamic range and imaging rate of SiPM detectors

The single photon response of a SiPM consists of a rapid (sub-nanosecond) rise followed by a much slower multi-exponential decay^13^. The first term of the decay is the overall capacitance of all avalanche diodes in the array discharging through the output resistance or transimpedance amplifier. As in a conventional photodiode or PMT, the time constant on this term can be minimized by using either a small output resistance, a transimpedance amplifier with a high gain-bandwidth product, or a smaller detector with a lower total capacitance. The second term is unique to SiPMs, and is formed by the capacitance of an individual avalanche diode recharging through its internal quenching resistor. Consequently, this term has the same duration as the recharge time for an individual SiPM, which is on the order of tens of nanoseconds. While AC coupling can remove this slow component, it results in drift of the signal zero point, making it unsuitable for imaging^14^. Conversely, imaging using the slow component results in higher gain, but also much lower maximum imaging rates due to a lowerbandwidth^15^. As a result, neither option is satisfactory for high speed imaging.

**Figure 1:**
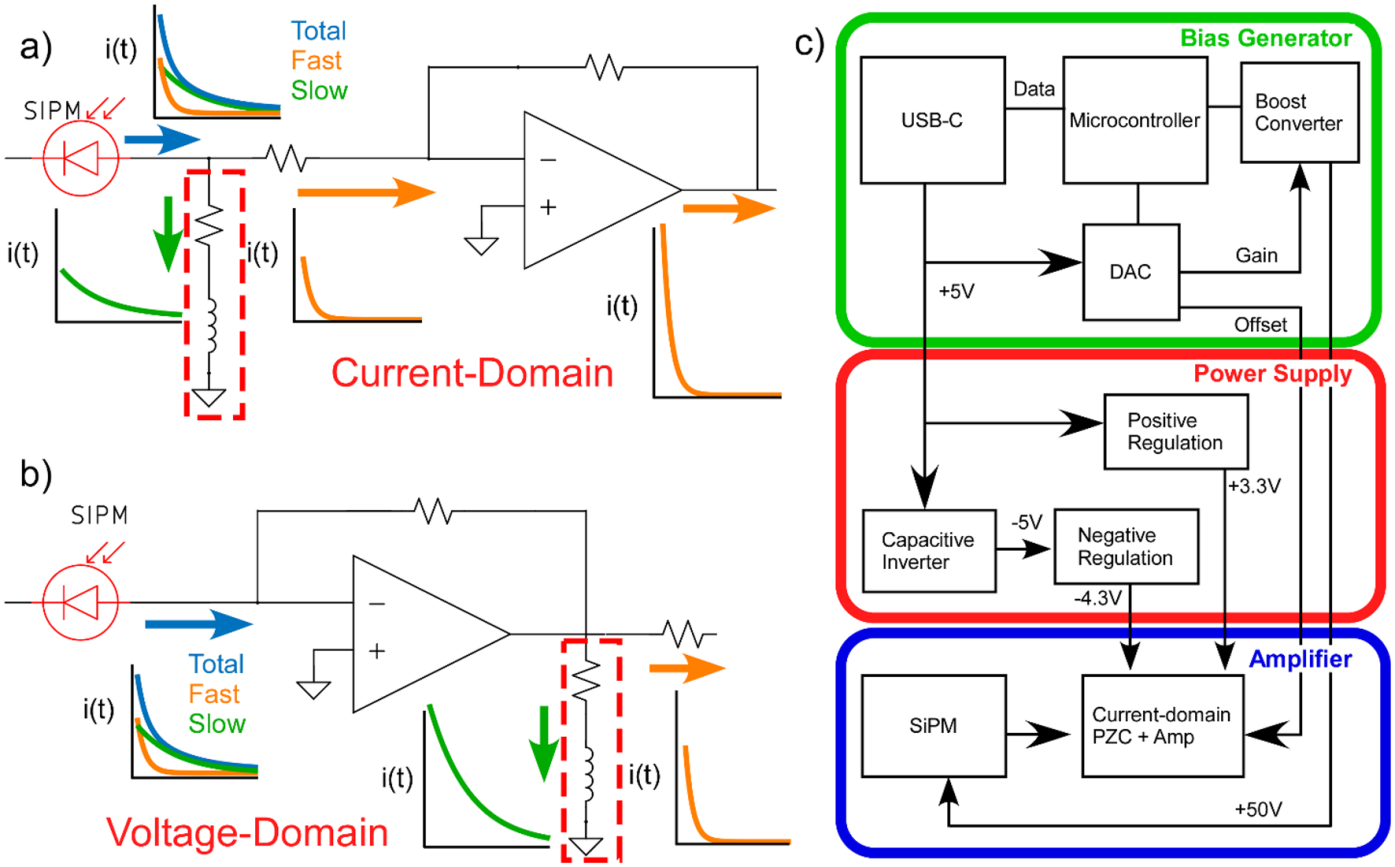
Pole zero cancellation schematics. (**a**) Proposed current-domain pole zero cancellation. The SiPM generates a multiexponential signal containing a small fast component, and a ten-fold larger slow component. A current divider with a series inductor (boxed component) shunts the larger slow component to ground while passing the fast component to the amplifier. (**b**) Conventional voltage-domain transimpedance amplifier. The entire multiexponential signal is amplified, and then after amplification the amplified slow component is removed by the inductor, wasting most of the amplifier dynamic range. (**c**) Block diagram of the current-domain PZC with bias generator and power supply.

A solution to extend the bandwidth of a SiPM detector beyond the limit imposed by the recharge time is to use the pole zero cancellation (PZC) method^14^. Briefly, this introduces a zero in the amplifier transfer function at the same frequency as the pole formed by the cell recharge time, ideally canceling it out and transmitting only the faster component of the SiPM response. In our prior work, we implemented the PZC method using a capacitive filter following a transimpedance amplifier, as has been described previously^14^. While intuitive, this solution required a complex circuit and could not utilize the dynamic range of the SiPM due to saturation of the transimpedance amplifier. Recognizing that the SiPM impulse response contains both a fast current pulse (the signal of interest) and a much larger recharge current (the slow component attenuated by the PZC), the dynamic range of a SiPM amplifier can be extended by performing PZC in the current-domain. By eliminating the larger slow component prior to the transimpedance stage, the entire amplifier dynamic range can be used to amplify the fast current component (Fig. 1), improving dynamic range by approximately an order of magnitude while enabling higher transimpedance bandwidths.

**Figure 2:**
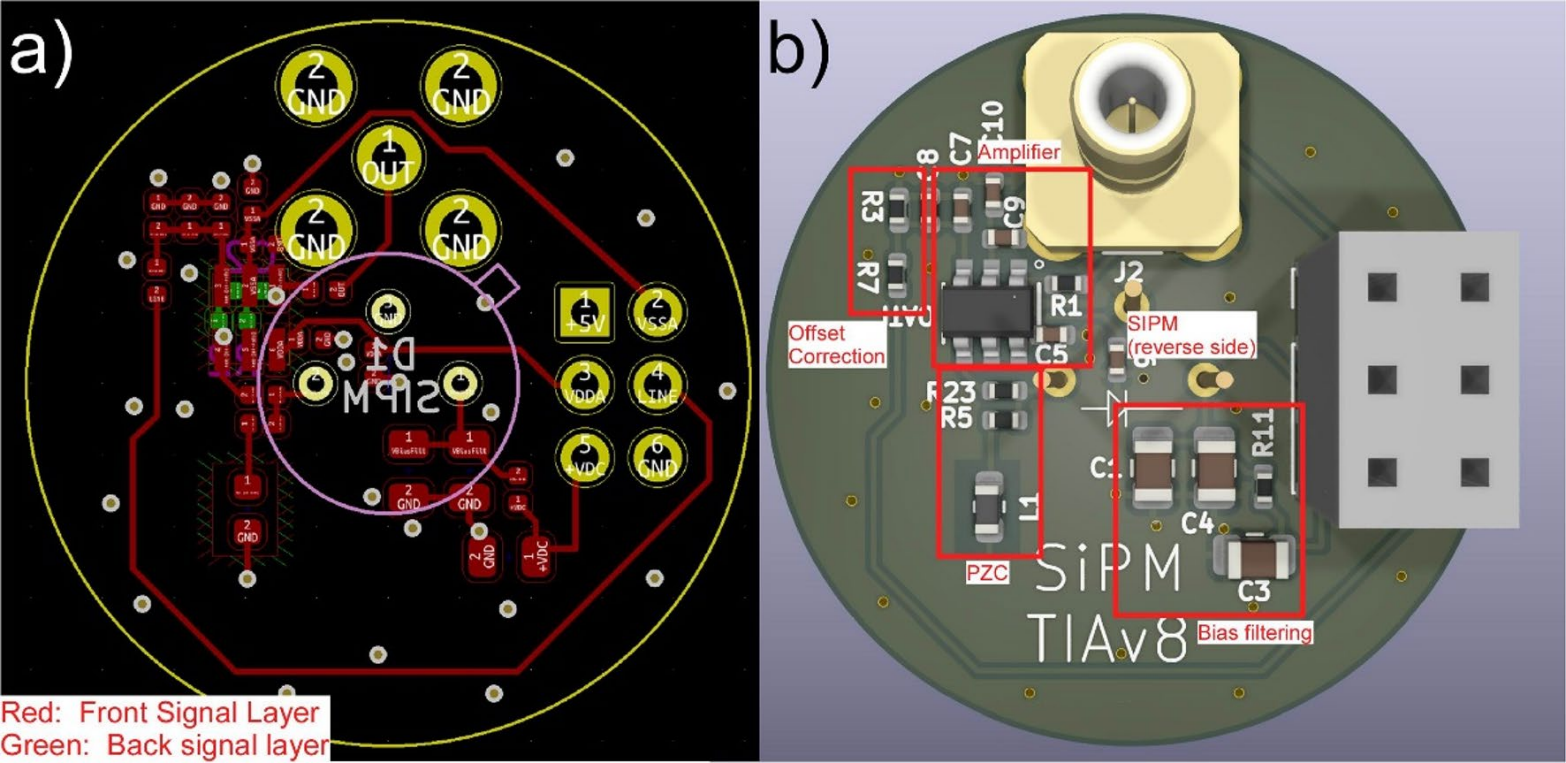
High dynamic range SiPM amplifier design. (**a**) Signal traces for the amplifier PCB. The bias voltage is filtered by a second order RC filter whose cutoff frequency depends on bias voltage but attenuates the 1 MHz switching frequency of the boost converter by more than 70 dB at 50v. A Hamamatsu S14420 is mounted on the reverse side. (**b**) Amplifier board with annotated component groups.

The current-domain PZC was implemented on a 2-layer PCB using a single OPA847 (Texas Instruments Inc.) as the amplifier. In contrast to the previous design, no secondary amplifier is required. In addition, we introduce a digitally controllable DC offset correction circuit, enabling the amplifier offset to be removed, or the amplifier output to be offset above zero. This allows both the positive and negative rails to contribute to the amplifier dynamic range. The entire detector module fits on a single 1-inch diameter PCB (Fig. 2).

### Custom high voltage bias generator

**Figure 3:**
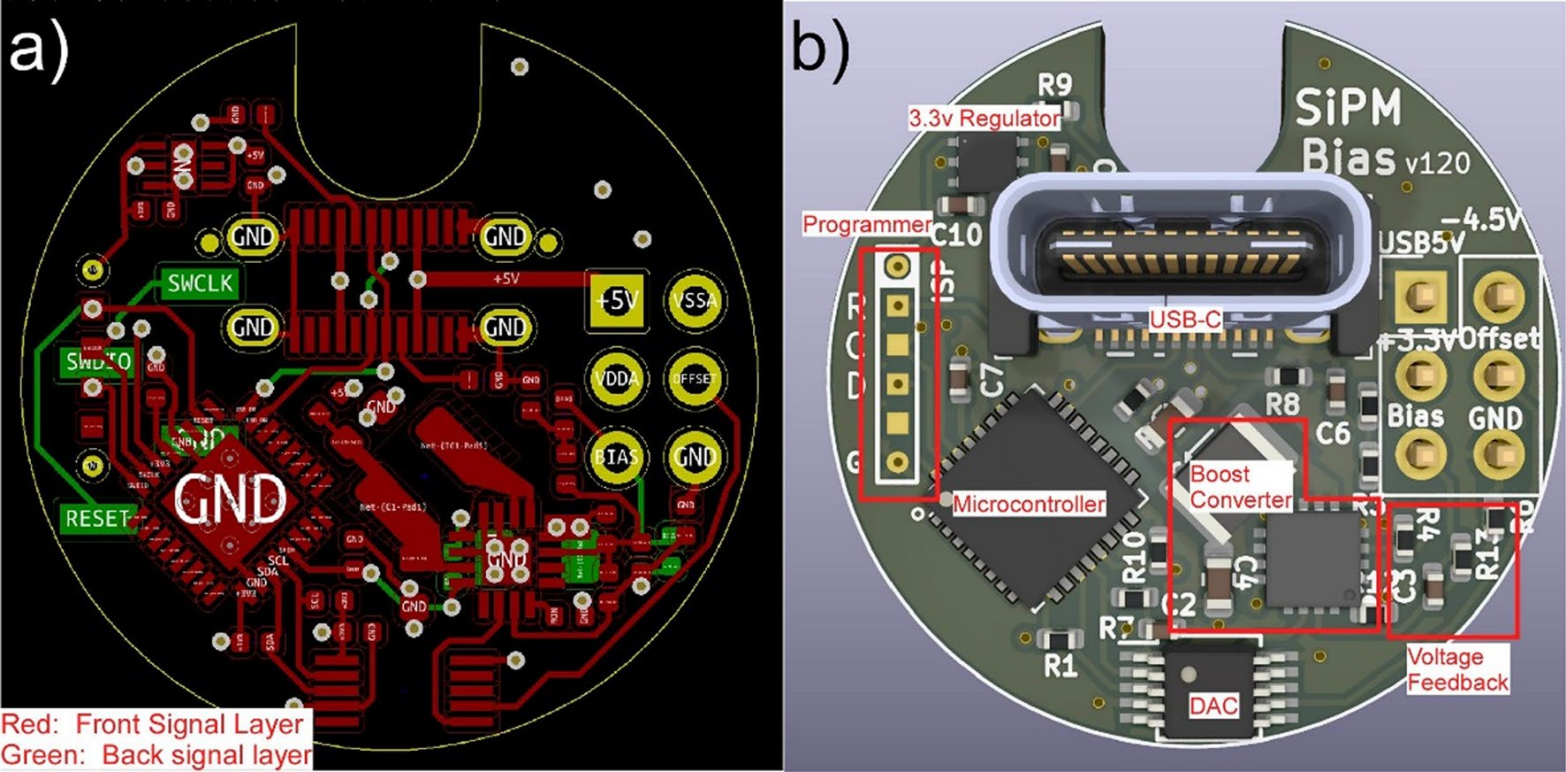
SiPM bias generator design. (**a**) Top (red) and bottom (green) signal layers for the bias generator PCB. Additional power and ground layers (internal) consisting of simple copper planes are omitted. (**b**) Assembled board with annotated functional components. A USB-C connector provides +5 V power, which is regulated down to 3.3 V for use by a microcontroller, which also provides the USB interface. A multichannel DAC is used to set the reference voltage for both the boost converter and the attached transimpedance amplifier.

Utilizing the dynamic range of a SiPM requires a bias supply that can generate moderately high voltages (~ 50 V) at a sufficient current to keep the individual SiPM avalanche diodes charged. In addition, the bias voltage needs to be adjustable, low noise and should be generated from a reasonably common supply voltage (e.g. 5 V). To address these requirements, we selected a low noise boost converter and an Arduino-compatible Cortex-M0 microcontroller. USB-C is used both to receive commands for the boost converter and for external (5 V) power. A multichannel I2C DAC is used to adjust the reference voltage for the boost converter and additionally provides an adjustable DC-offset for the amplifier output. The microcontroller, boost converter and DAC are integrated onto a circular 1-inch diameter 4-layer PCB (Fig. 3). Due to careful optimization of the boost converter and output filter, the device can provide 10 mA of bias current into a 50 V load.

**Figure 4:**
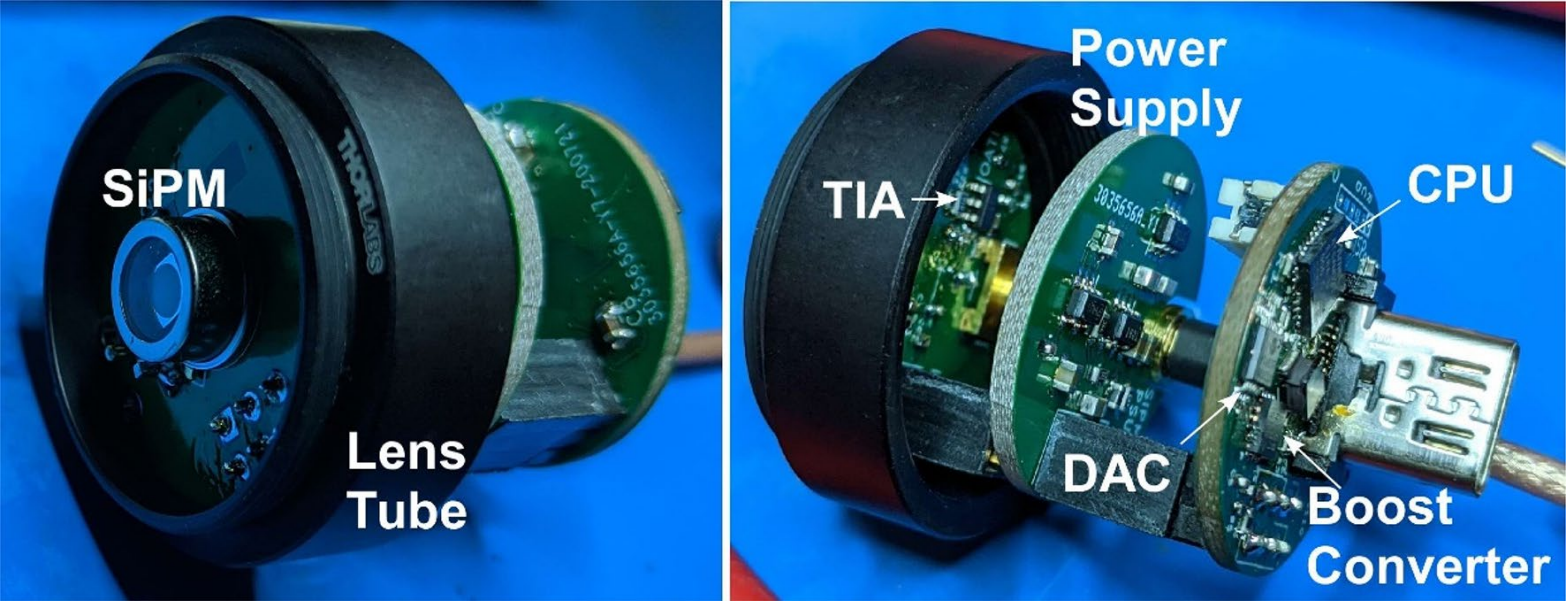
Assembled detector module in lens tube. The SiPM, current-domain PZC and TIA are on the front board, followed by a power supply board to generate the TIA voltage rails, and finally the boost converter and microcontroller on the last board.

The transimpedance and bias generator boards then stack together with an additional power supply board providing regulated positive and negative supply voltages to the amplifier and mount in an 1-inch lens tube, enabling the detector to be directly screwed into collection optics (Fig. 4). The total cost of each detector, including PCB fabrication, components, and S14420-3025 SiPM was $128.38 (see Supplementary material). Detailed assembly instructions and all design files are available under open source license at https://github.com/OpenSiPM/.

### Microscope system

The high dynamic range SiPM amplifier was installed in a custom-built two-photon microscope using a 24,000 line per second resonance scanner with a peak pixel rate of 78.0 MP/s and an average rate (accounting for oversampling due to resonant scanning trajectory) of 50.3 MP/s. Individual frames were 2048 × 2048 pixels. Illumination was provided by an 800 nm, 100 fs KMLabs Titanium:Sapphire (Ti:S) oscillator with a maximum power output of 275 mW and a repetition rate of 78 MHz. Thus, at the center of each frame, exactly one laser pulse was incident per pixel. In addition, the system had a Hamamatsu H10770-40PA GaAsP PMT with a transimpedance amplifier (PMT2101, Thorlabs, Inc.). 10× 0.45 NA (MRD70100, Nikon Instruments Inc.) and 16× 0.8 NA (MRP07220, Nikon Instruments Inc.) objectives were used for imaging. Except where noted, 25 MHz low pass filters were installed on both PMT and SiPM detectors to ensure equal bandwidth, and all imaging parameters were identical in all measurements. As in previous work^12^, the collection optics were designed such that the limiting aperture was inside the microscope objectives. Therefore the difference in active area of the SiPM (3 mm) and the PMT (5 mm) has no effect on light collection. Optical design files are provided along with the PCB designs.

### Human subjects research

Discarded and deidentified human tissue specimens were procured under a protocol approved by the Research Subjects Review Board at the University of Rochester Medical Center which waived the requirement for informed consent. All experiments were performed in accordance with the approved protocol, and all methods were performed in accordance with the relevant guidelines and regulations.

### Photon transfer curves

Photon transfer curves (PTCs) were calculated as described by Rice^16^. Briefly, the PTC relates the digital output of sensor to the number of photons received using the equality of the mean and variance of a Poisson process. For sensors such as 2D arrays with fixed integration periods, the pixel mean value can be plotted against the pixel temporal variance for increasingly illumination powers, with the slope being the detector gain. For the continuous-time analog output of a sensor, the calculation is more complex because the signal variance depends on both the sampling rate and the spectrum of the sensor transfer function. However, by calculating the equivalent analog bandwidth that has equal energy in the power spectrum, this dependence can be removed^16^. We calculated the PTC as shown in Eq. (1) where the sample mean and variance are the mean and variance of the analog to digital converter samples, the sampling rate is 125 MHz, and the equivalent bandwidth is the total integrated power in the device transfer function divided by the total power in an ideal brick-wall filter at one half the sampling rate (62.5 MHz). The actual value of the equivalent bandwidth depends on the effectiveness of the PZC circuit and the choice of lowpass filter, but was approximately 0.4, in good agreement with the 25 MHz nominal lowpass filter cutoff.

1 $${\raise0.7ex\hbox{${photons}$} \!\mathord{\left/ {\vphantom {{photons} s}}\right.\kern-\nulldelimiterspace} \!\lower0.7ex\hbox{$s$}} = {\raise0.7ex\hbox{${SampleMeanValue^{2} }$} \!\mathord{\left/ {\vphantom {{SampleMeanValue^{2} } {SampleVariance}}}\right.\kern-\nulldelimiterspace} \!\lower0.7ex\hbox{${SampleVariance}$}}*EquivilentBandwidth*SamplingRate$$

### Monte Carlo model

To understand the dynamic range of SiPM detectors and to validate our PTC calculations, we implemented a Monte Carlo (MC) model that simulates a SiPM receiving varying photon fluxes. Poisson-distributed photons arrive at random avalanche diodes in the SiPM array, generating a photocurrent proportional to the charge state of the APD. APDs are modeled as capacitors with the time-constants calculated from the Hamamatsu-specified quenching resistance and junction capacitance. The time of arrival of photons is recorded using a simulated 125 MHz sampling rate, identical to experiments. Using the mean and variance of the time-resolved photocurrent, PTCs were calculated from the simulated SiPM. For validation, PTCs generated using the MC model were used to calculate the detected photon flux which was then compared to the actual number of simulated photons launched. PTCs calculated from MC data were found to have greater than 99.5% agreement with the true number of photons launched.

## Results

### Bandwidth

**Figure 5:**
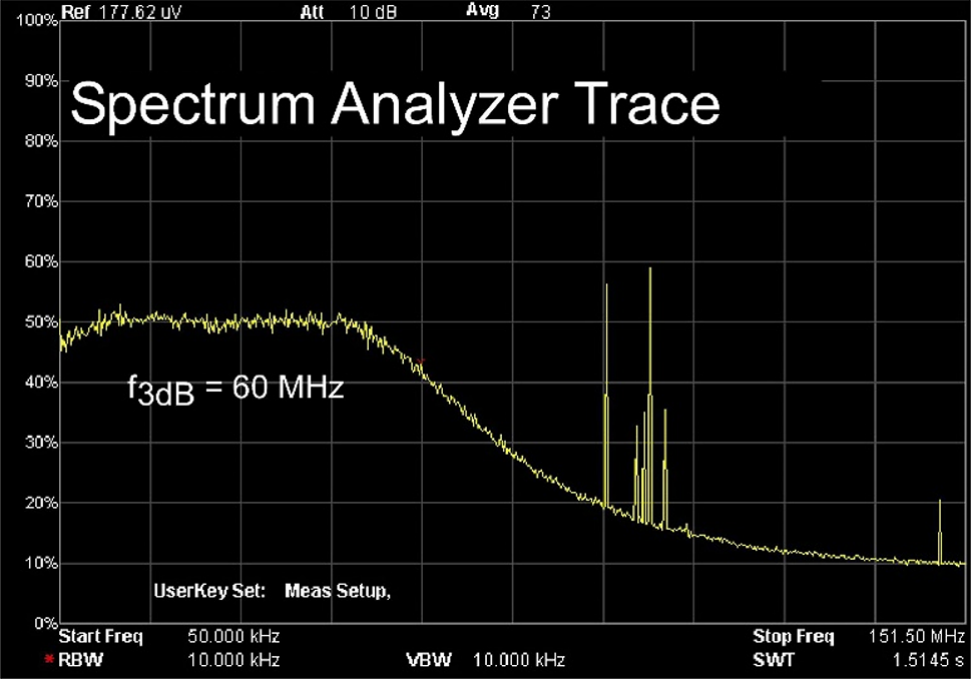
Spectrum analyzer trace of SiPM output. Electric spectrum analyzer trace of the SiPM output with low pass filter removed showing shot noise filtered by the system bandwidth. The transfer function is flat until 45 MHz and has a 3 dB bandwidth of 60 MHz due to the amplifier feedback capacitor. 90–110 MHz FM radio signals are detected by the unshielded detector, but have negligible total power compared to the dark noise and are removed by the low pass filter.

The current-domain PZC filter components were tuned to for optimal flatness by observing the power spectrum of shot noise using a Rigol DSA705 spectrum analyzer (Fig. 5). As designed, the detector has a 60 MHz 3 dB bandwidth, although this could be extended to greater than 75 MHz if the amplifier feedback capacitor and gain were reduced. We selected 60 MHz because it exceeds the maximum analog bandwidth of most practical scanning systems while minimizing FM radio interference. Following testing, external 25 MHz lowpass filters wereused in all other measurements on both SiPMs and PMTs to ensure equal bandwidth in all measurements.

### Dynamic range

**Figure 6:**
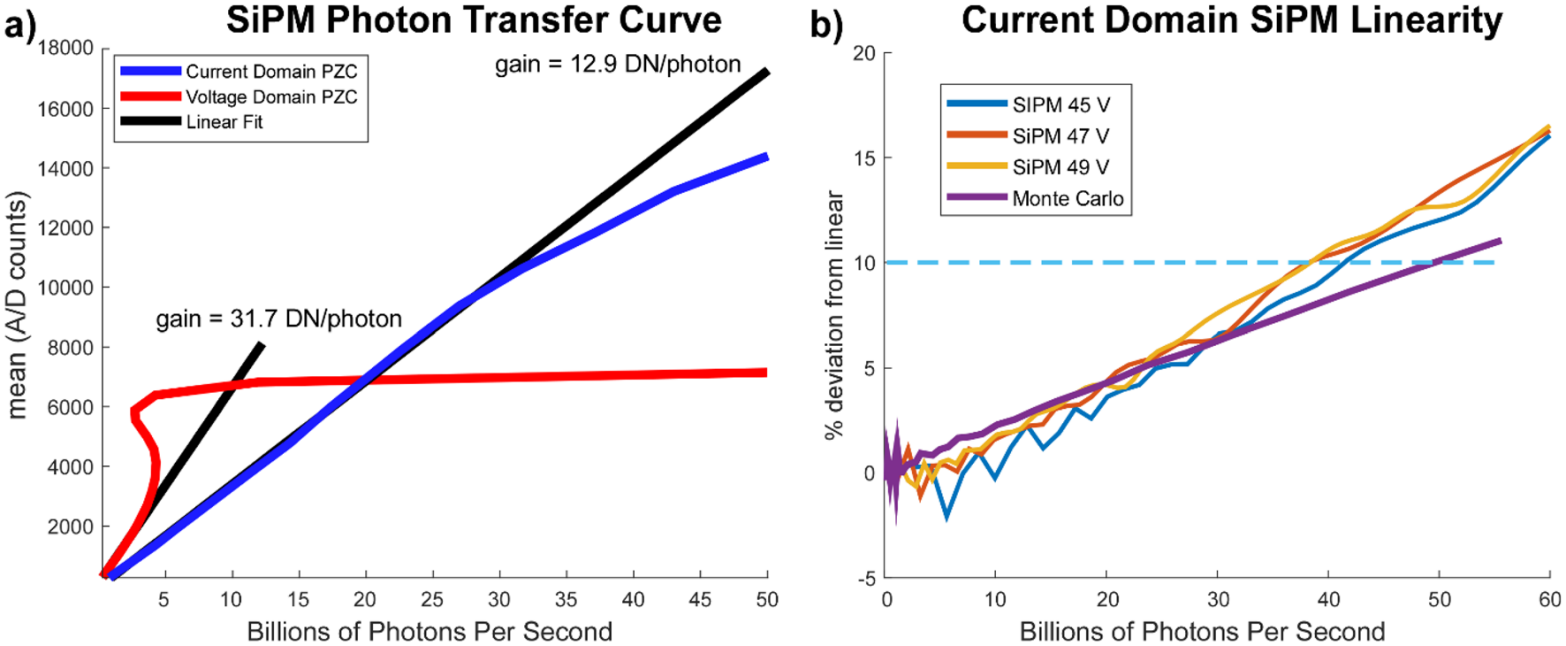
Dynamic range and linearity calculated from the device PTCs. (**a**) The original voltage domain PZC configuration saturates at 4 billion photons per second, while the improved current-domain PZC extends the 10% nonlinearity point to 40 billion photons per second. Both exceed the overcurrent protection circuit limit on the H10770 of approximately 3 billion photons per second. (**b**) PTC deviation from linear as a function of detected photons for the current-domain S14420-3025 at three bias voltages compared to simulated PTCs using the Monte Carlo model. Overall agreement is excellent until 30 billion photons per second, when the bias generator current limits begin to gradually drop the bias voltage. The oscillation in linearity is a measurement artifact due to minor ringing in the bias generator voltage in response to the square wave excitation and is most significant at lower gains due to proximity to the breakdown voltage.

PTCs were calculated for the original voltage-domain PZC amplifier^12^ and for the improved current-domain amplifier using an LED for illumination. Both detectors were operated at 7 V above breakdown (49 V). To avoid heating effects, which result in a minor shift in gain under sustained high photon flux, excitation was performed using a low duty cycle pulse train. In order to allow the SiPM array to reach equilibrium after excitation, the first 50 APD time constants worth of data were discarded. Using the gain calculated from the PTC, the variance was converted into photons per second. In contrast to the original design which was subject to oscillation above 4 billion photons per second, the current-domain PZC reaches a tenfold higher photon flux before gradually saturating due to a combination of current limits on the bias generator and SiPM cell saturation (Fig. 6a). In addition, the current-domain SiPM amplifier was compared to PTCs generated from the Monte Carlo model (Fig. 6b). Experimentally measured PTCs showed excellent agreement to simulated PTCs up to approximately 30 billion photons per second, above which the current limits on the bias generator and the slightly inhomogeneous distribution of light on the SiPM sensor result in increased nonlinearity relative to the Monte Carlo model. Depending on the bias voltage, the detectors reach 10% deviation from linear at 36–40 billion photons per second, although they can be used beyond 60 billion photons per second at the expense of some quantum efficiency. The gain of the current-domain amplifier was 12.9 DN/photon at 7 V above breakdown, while standard deviation of a dark frame was 5.97 DN at the same gain, or about 46% of the single photon amplitude. Thus, dark counts have a negligible effect on system noise.

### Maximum SNR

**Figure 7:**
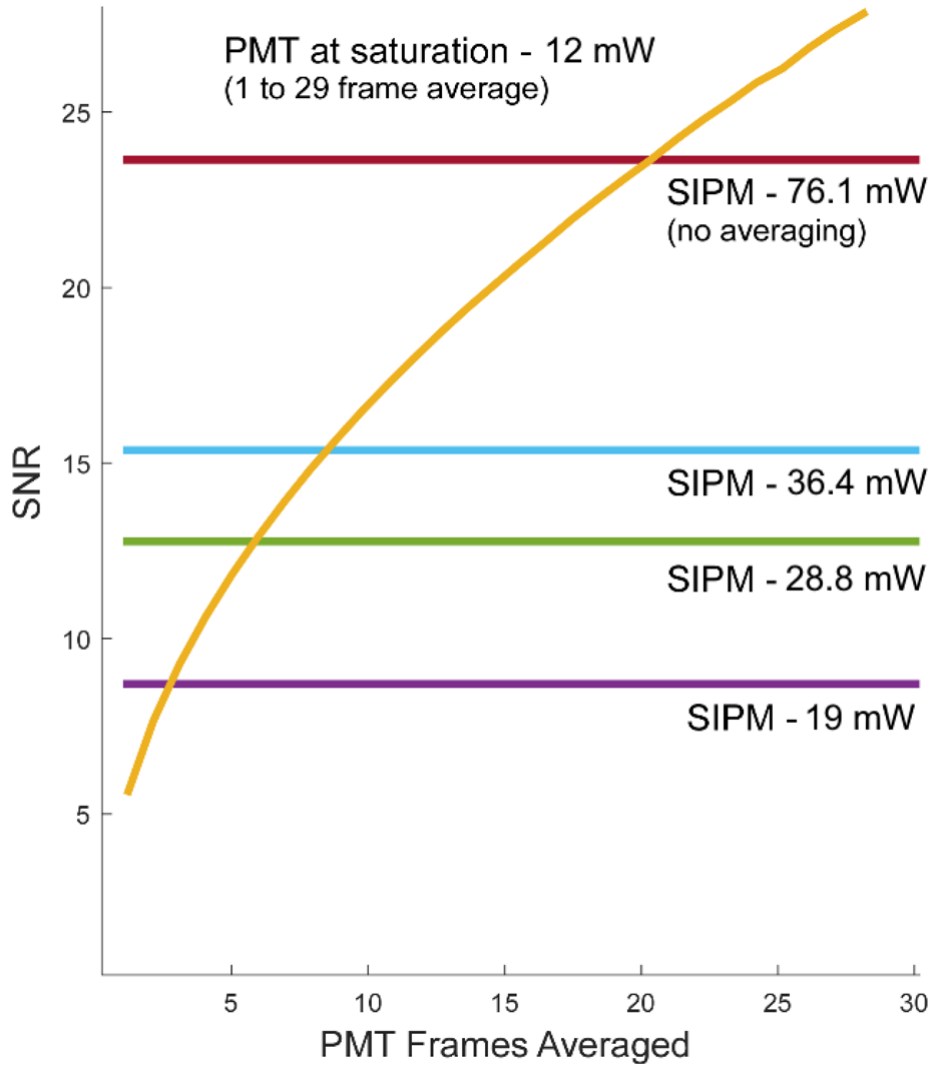
PMT SNR over number of frame averaging compared to single frame SiPM. Number of PMT frames that must be averaged to achieve equal SNR to single frame at four illumination powers. An H10770-40PA PMT customized with a 500 µA current limit was configured for the lowest gain (0.5 V control voltage) and the illumination power adjusted to the largest value that would not engage the overcurrent protection circuit. Both the SiPM and PMT were used to image a uniform fluorescent target, and the field of view was adjusted so that 1 laser pulse excited each pixel per frame. 8.5 individual PMT frames had to be averaged to match the SNR of a single SiPM frame acquired at 36.4 mW, while 19 were required for 76.1 mW.

We first tested the high dynamic range SiPM amplifier against the H10770-40PA GaAsP PMT. Both detectors were sequentially used to image a weakly fluorescent test slide (2273-R, Ted Pella Inc.). For the SiPM, sequential frames were acquired, and then the power was gradually increased until the entire laser power was incident on the sample. For the PMT, which saturated and triggered the overcurrent protection above 12 mW onto the sample, sequential images were acquired just below saturation. The per pixel SNR was calculated as the ratio of the mean to standard deviation of the pixel intensity at the center of the FOV where each pixel receives a single femtosecond pulse per frame. Figure 7 shows the PMT SNR as a function of number of frames averaged in comparison to single, unaveraged SiPM frames at various illumination powers. As expected, SNR improves with the square root of the number of frames averaged, and for lower SiPM illumination powers, the square of illumination power. Above approximately 42 mW, the fluorescent signal begins to saturate due to the finite number of fluorophores per voxel in the fluorescent test slide. Thus, for equal SNR, and avoiding saturation, the SiPM is 8.5 times faster (Table 1) when imaging this particular target, although higher rates are possible if fluorophore saturation is allowed.

**Table 1:** Imaging parameters for Fig. 7.

	Average power (mW)	Relative power	Pulse energy (nJ)	Total energy per pixel (nJ)	Relative imaging rate
SiPM	76.1	6.3	0.98	0.98	19
	36.4	3.0	0.47	0.47	8.5
PMT	12	1	0.15	2.8	1

### Tissue imaging

**Figure 8:**
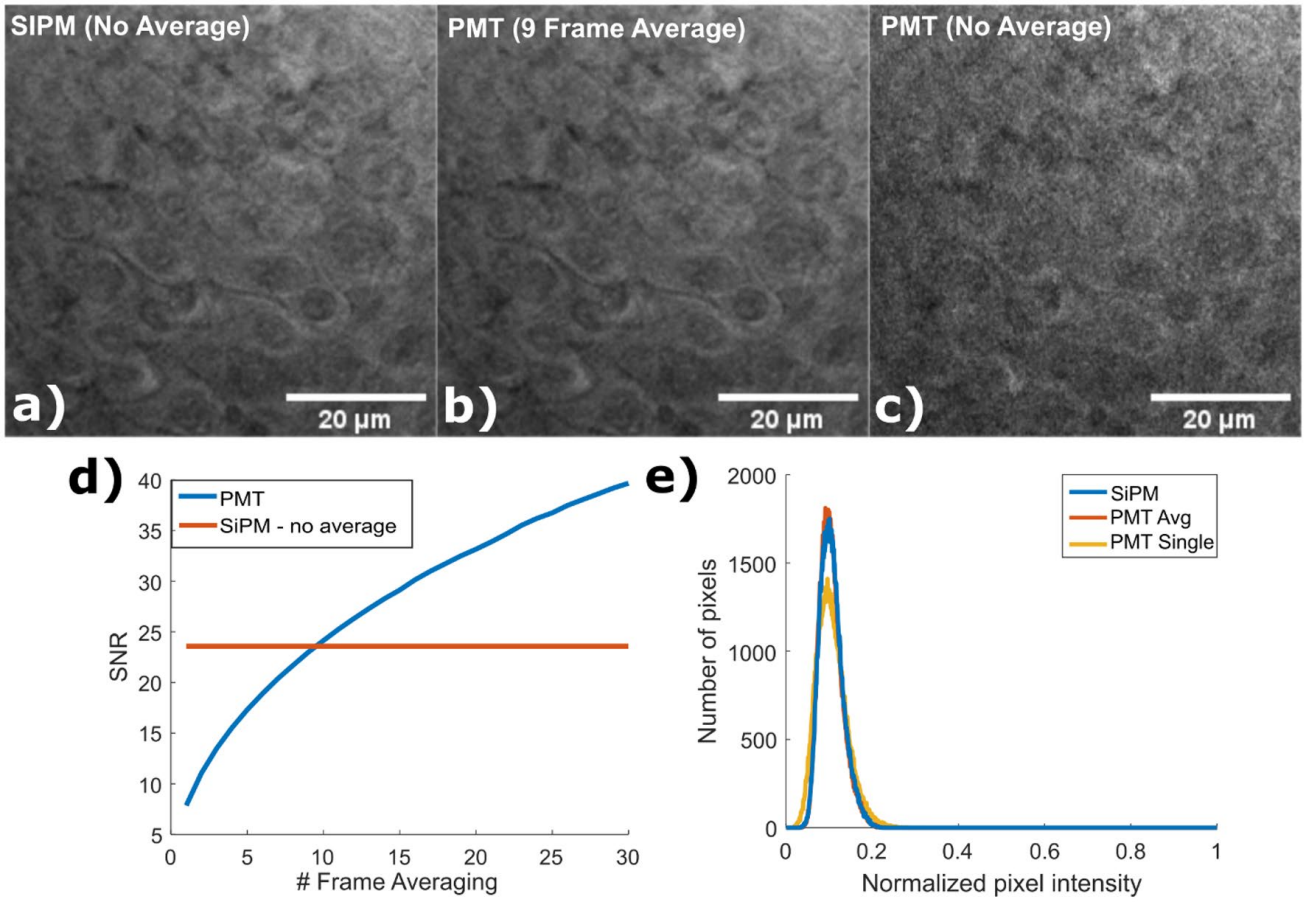
Comparison of SNR between the SiPM and PMT detectors. At maximum laser power, individual SiPM image frames (**a**) have similar SNR to 9.5 averaged PMT frames when operated just below the level that triggers the overcurrent protection circuit (**b**). In comparison, individual PMT frames have substantially more shot noise due to the low saturation power of the PMT (**c**). (**d**) SNR, defined as the mean pixel value divided by the standard deviation of pixel value over sequential frames, was calculated as a function of average number, giving a per frame SNR of 7.851 for the PMT and 23.574 for the SiPM. (**e**) Histogram of the pixel intensities for the image frames in (a), (b), and (c).

Next, we labeled human skin tumor specimens with 500 µg/ml of sulforhodamine 101, a stain that rapidly labels cytoplasm and nuclei of live cells. For the SiPM (Fig. 8a), the full ~ 270mW (~ 75 mW onto the sample) of laser power was used as we were not able to obtain saturation with our limited laser power. As before, power for the PMT was adjusted to just below triggering the overcurrent protection (Fig. 8c). Multiple frames were acquired using both the SiPM and PMT, and the per pixel SNR calculated from the temporal variation in pixel intensity. Sequential PMT frames were then averaged and the SNR calculated as a function of average number for the PMT (Fig. 8d), indicating that 9.5 PMT frames (Fig. 8b) needed to be averaged to obtain the same SNR and histogram (Fig. 8e) as a single SiPM frame.

**Figure 9:**
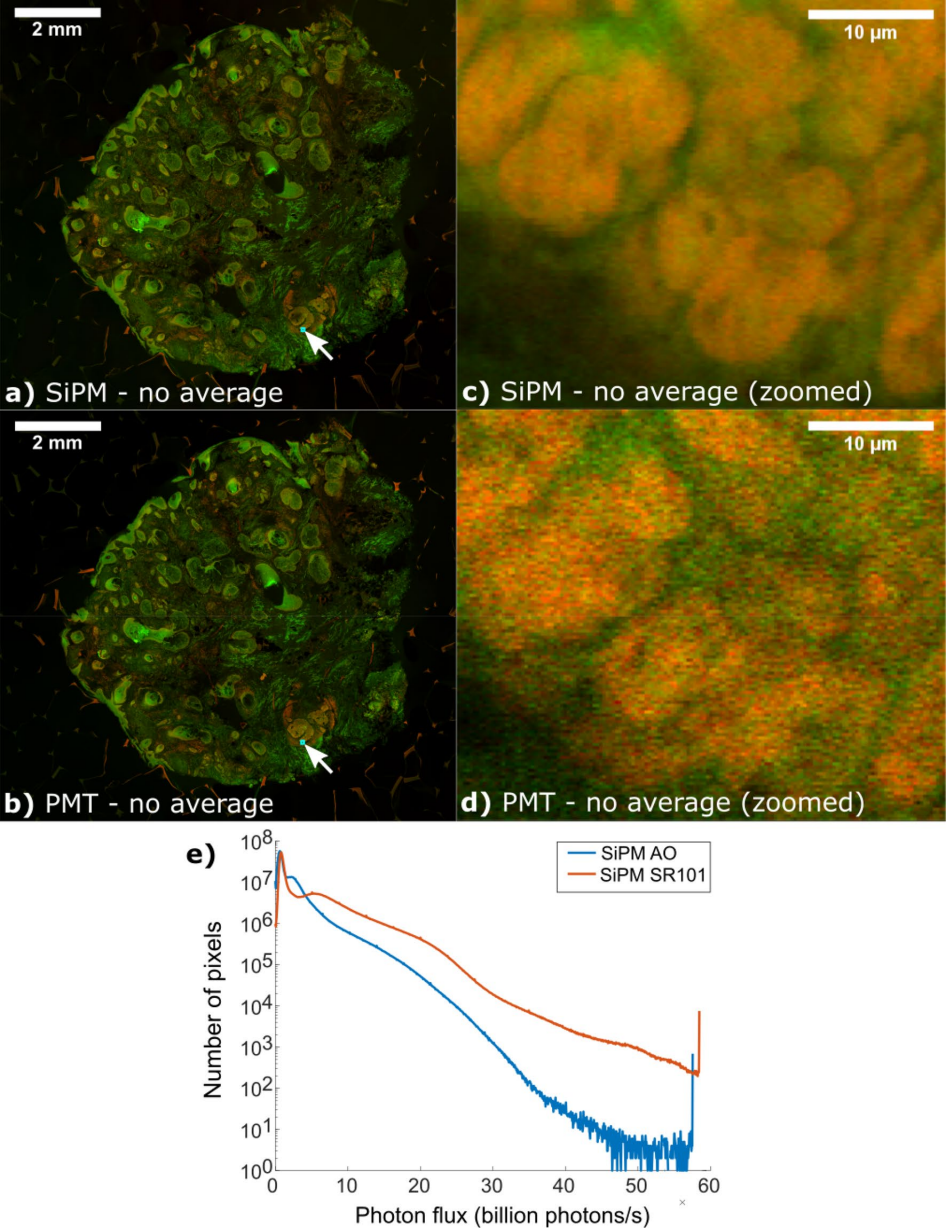
SiPM vs PMT tissue specimen mosaic comparison. Comparison mosaics between (**a**) SiPMs and (**b**) PMTs using an AO and SR101 labeled surgical specimen acquired at 50 MP/s. Zoomed views (**c**, **d**) of the blue box region show basal cell carcinoma. In contrast to the PMT, the SiPM has dramatically higher SNR due to much higher photon counts enabling visualization of sub-nuclear features in carcinoma cells (**c**) that are obscured by shot noise in (**d**). Histogram values of the per pixel photon flux for the SiPM (**e**) showing the distribution of photon detection rates in (**a**). Note that generating a similar histogram for the PMT is not possible because excess noise prevents calculation of the PTC for the PMT. Full resolution link: https://imstore.circ.rochester.edu/papers/sipm2020/sipmVsPmtMosaic.html.

Finally, we labeled additional tumor specimens with 500 μg/ml of sulforhodamine 101 and 500 μg /ml of acridine orange, a dye that stains DNA. Two channel imaging at 16×/0.8NA was performed using a matched pair of H10770-40PA PMTs and a matched pair of S14420-3025 SiPMs. Tissue specimens were sequentially imaged with the PMTs at the lowest gain setting (to maximize dynamic range as described previously^12^) using the highest illumination power that did not trip the overcurrent protection. SiPM illumination power was increased until fluorescence deviated from the expected intensity-squared relationship for unsaturated two-photon excitation by more than 10%, resulting in an illumination power of 40 mW. No averaging was used for either detector. Mosaics of the entire specimens were recorded. Figure 9 shows significantly higher SNR for the SiPM images, with sub-nuclear features in the carcinoma easily visible on the SiPM image (Fig. 9c). In comparison, for the PMT image, excess and shot noise obscures much of the sub-nuclear detail (Fig. 9d). Finally, histogram analysis of the image pixels (Fig. 9e) demonstrate that the dynamic range of the SiPM is being utilized.

### Effect of laser repetition rate

**Figure 10:**
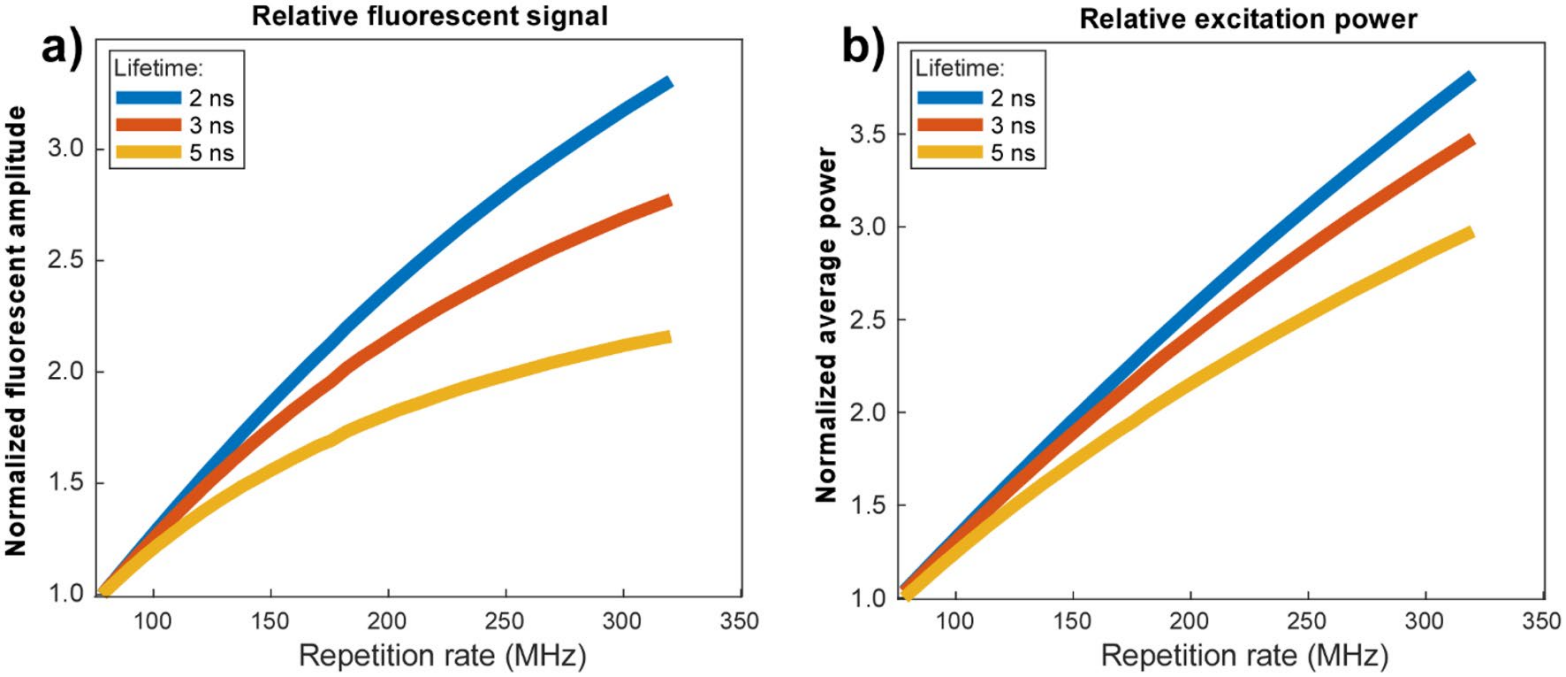
Fluorescent yield of fluorophores over laser repetition rate. Relative fluorescent yield per second (**a**) and average excitation power required (**b**) to keep 10% of fluorophores in a pixel excited in steady state for 3 different single exponential fluorescent lifetimes. Amplitudes are normalized with 78 MHz equal to 1.0. For shorter lifetimes, both fluorescent yield and excitation power are nearly linear, with a fourfold increase in repetition rate yielding a 3.3-fold increase in fluorescence at 3.8 times the average power. For longer lifetimes, fluorophores do not have time to fully relax between excitations, and pulse energy must be decreased to keep a constant 10% of fluorophores excited, resulting in both lower fluorescent yield per time and lower maximum average excitation power.

To determine how laser repetition rate could be adjusted to maximize fluorescent signal, we simulated exciting fluorophores with various single exponential fluorescent lifetimes. At each laser repetition rate, we solved numerically for the average fluorescent photon flux (Fig. 10a) generated by exciting 10% of fluorophores and for the average laser power required (Fig. 10b). The results strongly depend on fluorescent lifetime, but in all cases repetition rates substantially higher than the common 80 MHz repetition result in much higher signal without saturation.

## Discussion

Compared to techniques such as light sheet microscopy which use parallel detection of photons across millions of individual detectors to obtain detector saturation powers on the order of trillions of photons per second, the imaging speed and maximum shot-noise limited SNR of most laser scanning microscopes is much lower. However, point scanning enables higher resolution and deeper tissue imaging due to the reduced sensitivity to light scattering, forcing an unfortunate tradeoff between resolution, imaging speed and SNR, with two-photon imaging rates typically having poor SNR above a few million pixels per second. The introduction of SiPMs, which parallelizes tens of thousands individual photon counters, enables some of the dynamic range and quantum efficiency advantage of array sensors to be applied to point scanning, resulting in higher imaging rates and SNR than what is possible using conventional PMTs. In our previous work, we demonstrated that SiPMs have higher sensitivity than state-of-the-art GaAsP PMTs due to their high quantum efficiency and negligible excess noise factor, resulting in higher SNR at constant excitation power. In this work, we have demonstrated that improved detection electronics enable more than an order of magnitude increase in photon throughput while retaining single photon sensitivity. This improvement in throughput enables proportional increases in maximum imaging rates or SNR. Furthermore, compared to high speed sCMOS image sensors or photomultiplier tubes costing many thousands of dollars, our entire detector module costs less than $130, making it extremely cost-effective, especially for multiplexing spectral channels.

While the parallel detection of SiPMs enables photon detection rates approaching CMOS image sensors, the need to sequentially excite pixels still imposes a lower practical detection rate than techniques such as light sheet microscopy. Specifically, the maximum fluorescent signal cannot exceed the product of the number of ground state fluorophores per voxel, the quantum yield and the laser repetition rate. Increasing the laser repetition rate will enable each molecule to be excited more frequently but is ultimately limited by the fluorescent lifetime (several nanoseconds for typical fluorophores). Figure 10 shows simulations of the fluorescent signal and required excitation power as a function of laser repetition rate if the pulse energy is adjusted to keep 10% of fluorophores in the excited state. While the optimal repetition rate depends on the exact fluorescent lifetime, using 3–4 times the typical 80 MHz Ti:S rate (as can be provided by cost-effective fiber laser systems) would enable 2–3 times higher pixel rate at equal SNR for typical fluorophores. In our measurements, we observed saturation beginning around 40 mW average power onto the sample (0.5 nJ pulse energy) for labeled human tissue specimens. These results suggest that photon fluxes approaching 200 billion photons per second may be possible using single point scanning for densely labeled specimens (e.g. cleared tissue specimens) using conventional resonant scanning. Samples can also be pushed further into saturation which results in significantly higher SNR with the caveat that this will degrade the point spread function by enabling two-photon events further from the beam focus.

Alternatively, higher imaging rates could be obtained through multiple point scanning. Previous literature has demonstrated multifocal imaging using 6.7 ns^17^ and 3 ns^18^ inter-pulse delay for each scanning point with acceptable crosstalk. This approach could be directly applied to double or quadruple the 50 MP/s average rate demonstrated here to 100–200 MP/s by scanning two or four points offset in time with little or no loss of SNR. Further increases in imaging speed could be obtained by spatially multiplexing multiple SiPM detectors in each spectral channel or using SiPMs with more parallel APDs to extend dynamic range. Alternatively, these approaches could allow higher imaging speeds to be used for more weakly fluorescent samples than would otherwise be possible.

The use of high average powers raises the prospect of photodamage through both one-photon and two-photon processes. As demonstrated in Table 1, the use of high average powers without averaging results in significantly less energy deposition per pixel than the conventional approach of averaging multiple excitations due to the intensity-squared dependence of two-photon absorption. Therefore, if higher average powers are combined with higher pixel rates, the risk of one photon damage is decreased. Conversely, for two-photon processes, photobleaching increases more rapidly than fluorescent yield with increasing pulse energy^19–21^ suggesting that photobleaching may be a more significant limitation. However, the probability of photobleaching a specific molecule per excitation is usually negligible, and so measurable photobleaching typically requires many excitations. Accordingly, we observed that if we did not drive the specimen into saturation, we could image voxels more than 100 times at 1 pulse per voxel without observing a measurable decrease in fluorescence, as expected because each molecule is only excited a few times.

As demonstrated in Fig. 6, the current design comes close to utilizing the full dynamic range of the 25 μm SiPM microcells used in the S14420-3025 SiPM but is ultimately limited by the maximum photocurrent that the bias generator can supply. While improving the bias generator would lead to only a minor improvement in dynamic range for this specific SiPM, newer models utilize smaller microcells with more rapid recharge times, enabling dynamic ranges in excess of 100 s of billions of photon/s. Further improvements in power supply and amplifier design will be required to utilize these extremely large dynamic range detectors.

Previous work has used SiPM detectors for biomedical applications^15,22–24^. While these publications suggested the potential of the technology, most have used readout circuits adopted from LIDAR or other fields due to the complexity of optimizing SiPM electronics for microscopy and the lack of suitable commercial products. As a result, maximum bandwidth, dynamic range, sensitivity and/or imaging rates have been substantially limited, obscuring the full potential of SiPM detectors to enable high speed and high sensitivity imaging. To enable access to more optimal SiPM detectors, we have formed the OpenSiPM project, a collaborative open source project that serves as a repository for the designs presented here and for future improvements in detector electronics. A new generation of SiPM detectors are now available with dynamic ranges extending into the hundreds of billions of photons per second. Further improvements in detection electronics may allow for large future improvements in scanning speed, particularly for parallel point scanning or high laser repetition rate imaging.

## Conclusion

We have demonstrated an extremely low-cost design for a photodetector module based on a novel approach to silicon photomultiplier read out that enables more than an order of magnitude increase in maximum photon detection rates as compared to conventional photomultiplier tubes. We show that this increase in dynamic range, combined with the lower excess noise and higher sensitivity of SiPMs, directly translates into higher SNR and higher imaging rates than are possible with photomultiplier tubes. Our designs are freely available, and combined with the higher sensitivity of SiPMs, can enable dramatically higher imaging speeds, higher SNR, and much lower costs in applications such as two-photon or confocal microscopy. For many high-speed fluorescence imaging instruments, SiPMs are a superior choice to conventional PMTs.

## Supplementary Information

Supplementary Information.

## Data Availability

Links to full resolution data of all mosaicked images presented are provided in the figure captions. Design files and Monte Carlo source code are available on https://github.com/OpenSiPM/.
