# Publisher Correction: Ultrahigh-speed point scanning two-photon microscopy using high dynamic range silicon photomultipliers

**DOI:** 10.1038/s41598-021-88210-x

**Published:** 2021-04-13

**Authors:** Vincent D. Ching-Roa, Eben M. Olson, Sherrif F. Ibrahim, Richard Torres, Michael G. Giacomelli

**Affiliations:** 1 Department of Biomedical Engineering, University of Rochester, 207 Goergen Hall, BOX 270168, Rochester, NY 14627 USA; 2 Department of Laboratory Medicine, Yale University, New Haven, CT USA; 3 Department of Dermatology, University of Rochester Medical Center, Rochester, NY USA; 4 Rochester Dermatologic Surgery, PC, Victor, NY USA

Correction to: *Scientific Reports* https://doi.org/10.1038/s41598-021-84522-0, published online 04 March 2021.

This Article contains an error in Figure 9 where due to a technical error a white line is visible across panels b and d. The correct Figure 9 appears below as Figure 1.

**Figure 1:**
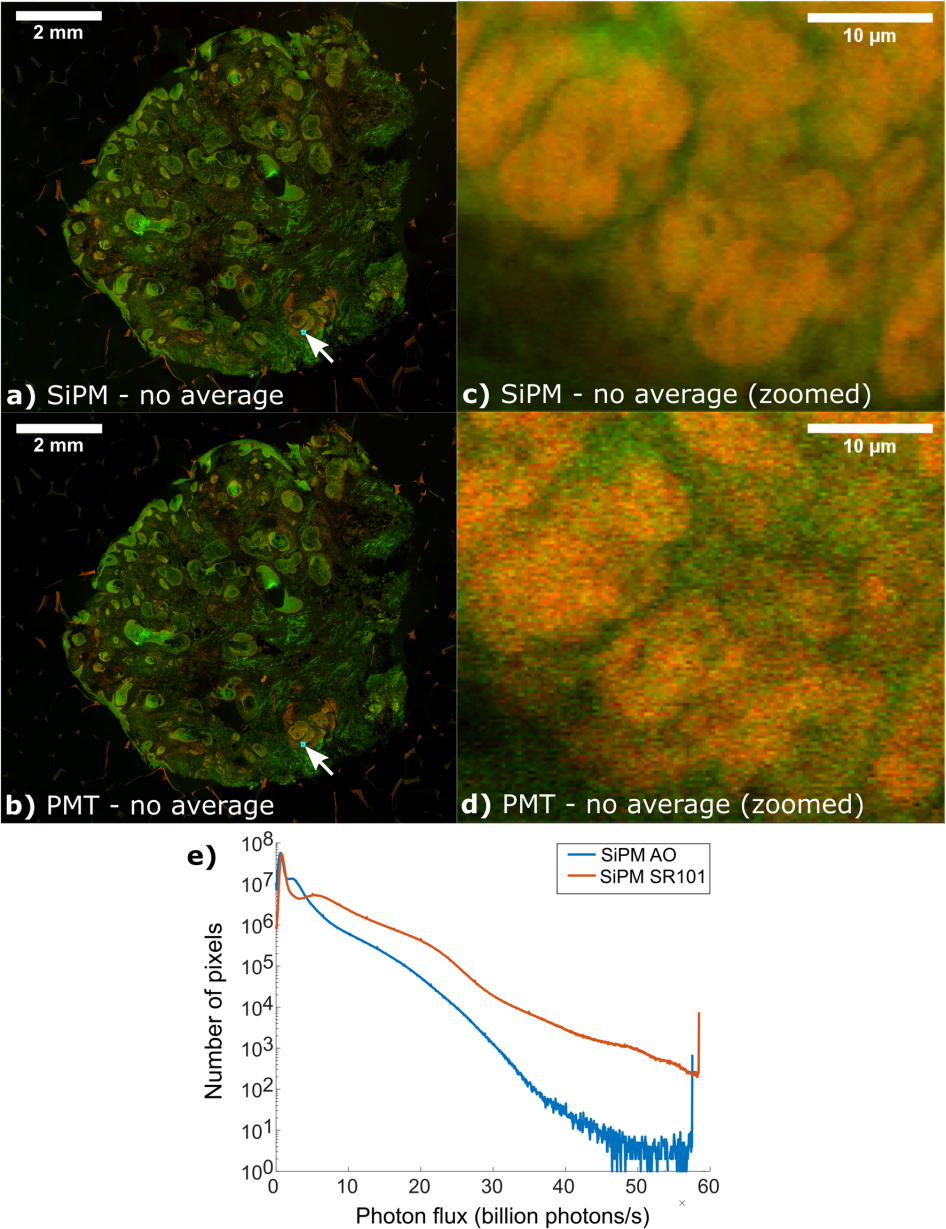
SiPM vs PMT tissue specimen mosaic comparison. Comparison mosaics between (**a**) SiPMs and (**b**) PMTs using an AO and SR101 labeled surgical specimen acquired at 50 MP/s. Zoomed views (**c**,**d**) of the blue box region show basal cell carcinoma. In contrast to the PMT, the SiPM has dramatically higher SNR due to much higher photon counts enabling visualization of sub-nuclear features in carcinoma cells (**c**) that are obscured by shot noise in (**d**). Histogram values of the per pixel photon flux for the SiPM (**e**) showing the distribution of photon detection rates in (**a**). Note that generating a similar histogram for the PMT is not possible because excess noise prevents calculation of the PTC for the PMT. Full resolution link: https://imstore.circ.rochester.edu/papers/sipm2020/sipmVsPmtMosaic.html.

